# Investigation and Evaluation of the Transdermal Delivery of Ibuprofen in Various Characterized Nano-Drug Delivery Systems

**DOI:** 10.3390/pharmaceutics15102413

**Published:** 2023-10-03

**Authors:** Jeanri Myburgh, Wilna Liebenberg, Clarissa Willers, Admire Dube, Minja Gerber

**Affiliations:** 1 Centre of Excellence for Pharmaceutical Sciences (Pharmacen™), North-West University, Potchefstroom 2531, South Africa; jeanri.meesters@gmail.com (J.M.); wilna.liebenberg@nwu.ac.za (W.L.); clarissawillers@gmail.com (C.W.); 2 School of Pharmacy, Faculty of Natural Sciences, University of the Western Cape, Cape Town 7535, South Africa; adube@uwc.ac.za

**Keywords:** ibuprofen, NSAIDs, nano-emulsion, nano-emulgel, colloidal suspension, transdermal drug delivery, in vitro cytotoxicity

## Abstract

The aim was to assess the suitability of three nano-based transdermal drug delivery systems containing ibuprofen: a nano-emulsion, a nano-emulgel, and a colloidal suspension with ibuprofen-loaded nanoparticles. Understanding the transdermal delivery of ibuprofen using nano-based drug delivery systems can lead to more effective pain relief and improved patient compliance. Characterization tests assessed the suitability of the developed drug delivery systems. Membrane release and skin diffusion studies, along with tape stripping, were performed to determine drug release and skin permeation of ibuprofen. In vitro cytotoxicity studies on HaCaT cells were conducted using MTT and neutral red assays to evaluate the safety of the developed drug delivery systems. Characterization studies confirmed stable drug delivery systems with ideal properties for transdermal delivery. Membrane release studies demonstrated the successful release of ibuprofen. In vitro skin diffusion experiments and tape stripping, detecting ibuprofen in the receptor phase, stratum corneum-epidermis, and epidermis-dermis, indicating successful transdermal and topical delivery. The in vitro cytotoxicity studies observed only minor cytotoxic effects on HaCaT cells, indicating the safety of the developed drug delivery systems. The investigation demonstrated promising results for the transdermal delivery of ibuprofen using the developed drug delivery systems, which contributes to valuable insights that may lead to improved pain management strategies.

## 1. Introduction

Chronic inflammatory pain is a significant global concern, as evidenced by research indicating that individuals around the world regularly seek medical care for this reason, but sufficient management thereof remains an unmet need [1,2,3,4]. Inflammatory pain is perceived due to the nociceptive processing of chemical mediators, such as prostaglandins, that are released by the inflammatory response [5,6,7]. The inflammatory response serves a necessary immunological function, but chemical mediators released by the inflammatory response aggravate pain; therefore, if chemical mediators are countered, pain can be diminished [8,9].

Non-steroidal anti-inflammatory drugs (NSAIDs) are regarded as the most effective pharmacological strategy that treats pain and inflammation by blocking the cyclooxygenase (COX)-2 enzyme, which inhibits the biosynthesis of prostaglandin [4]. However, non-selective NSAIDs (nsNSAIDs), such as ibuprofen, also inhibit the COX-1 enzyme responsible for producing prostaglandins linked to optimal physiological function, such as the protection of the gastrointestinal mucosa [10]. Ibuprofen is commonly used as monotherapy for the alleviation of pain and inflammation caused by musculoskeletal diseases such as rheumatoid arthritis and osteoarthritis; it is also regularly used to treat both chronic and acute injuries experienced by athletes [11]. Long-term oral usage of nsNSAIDs (such as ibuprofen) frequently leads to undesirable gastrointestinal side-effects (i.e., upper GI bleeding, ulceration, and dyspepsia) due to COX-1 inhibition; therefore, highlighting the need for alternative drug delivery routes, such as transdermal drug delivery [12,13]. Topically administering ibuprofen can bypass detrimental adverse effects associated with chronic oral intake while achieving targeted relief of pain and inflammation (i.e., muscles, joints, etc.) [11].

Although the skin is a convenient and accessible drug delivery route, active pharmaceutical ingredients (APIs) are required to have ideal physicochemical characteristics to permeate the complex and rate-limiting barrier, known as the stratum corneum, successfully [14,15]. If an API does not possess ideal characteristics, as in the case of ibuprofen, well-developed drug delivery systems can be utilized to achieve successful transdermal delivery [16,17,18]. Therefore, during this study, ibuprofen was included in a nano-emulsion, nano-emulgel, and a colloidal suspension with drug-loaded nanoparticles.

Nano-emulsions are kinetically stable isotropic systems of two immiscible liquids, commonly oil and water, with small droplet sizes (50–200 nm) that present useful properties for drug delivery such as increased surface area, stability, and permeation, resulting in higher bioavailability and therapeutic efficiency [19,20]. Evening primrose oil (EPO) was used as the oil phase and simultaneously served as a penetration enhancer due to its suitable range of attributes [21]. High energy emulsification, by means of an ultrasonicator, was applied to produce oil-in-water (o/w) nano-emulsions [22]. Nano-emulgels present all the desirable qualities of nano-emulsions with the benefit of easier application, higher viscosity, and increased stability due to the inclusion of a gelling agent [23]. Carbopol^®^ Ultrez 20 was the selected gelling agent for inclusion into the aqueous phase of the optimized nano-emulsion since it self-wets and disperses within minutes, providing uncomplicated use [24]. Colloidal suspensions have vast applications in the field of nanotechnology and hold a great deal of promise and interest in the transdermal delivery sector [25]. Drug-loaded nanoparticles were formulated using the emulsion-solvent evaporation technique and provide particle sizes of 1–100 nm with properties such as high stability, fewer side effects, an increased surface-to-volume ratio, and improved permeation through the skin [26,27,28,29]. Therefore, this study suspended ibuprofen-loaded nanoparticles in an aqueous phase, which acts as a carrier system, to obtain a colloidal suspension [30].

This study aimed to investigate and evaluate the transdermal delivery of ibuprofen when formulated in a nano-emulsion (NE), nano-emulgel (NEG), and colloidal suspension containing ibuprofen-loaded nanoparticles (CS). The developed drug delivery systems were characterized and compared against a market-related product (MP) using membrane release studies, in vitro skin diffusion studies, and tape stripping. Methyl thiazolyl tetrazolium (MTT) and neutral red (NR) assays also determined the potential in vitro cytotoxicity of the NE, a placebo of NE (PNE) and CS on human keratinocyte (HaCaT) cells.

## 2. Materials and Methods

### 2.1. Materials

DB Fine Chemicals (Johannesburg, South Africa) supplied ibuprofen (purity 99.91%), and the MP (5% ibuprofen) was purchased from a local pharmacy (Potchefstroom, South Africa). Excipients used to formulate the nano-emulsions (Tween^®^ 80, Span^®^ 60), the nano-emulgels (Carbopol^®^ Ultrez 20), and the nanoparticles (polycaprolactone (PCL), polyvinyl alcohol (PVA), and sucrose) were purchased from Sigma-Aldrich (Johannesburg, South Africa), while CJP Chemicals (Johannesburg, South Africa) provided EPO and LabChem (Johannesburg, South Africa) supplied the dichloromethane (DCM). Sigma-Aldrich (Johannesburg, South Africa) supplied potassium dihydrogen phosphate and sodium hydroxide to prepare the phosphate buffer solution (PBS), as well as the MTT, NR solution, Triton™ X-100, dimethyl sulfoxide (DMSO) and non-essential amino acids (NEAA) for the cytotoxicity studies. Associated Chemical Enterprises (Johannesburg, South Africa) supplied chromatography-grade acetonitrile, analytical-grade formic acid, and methanol. A Rephile Direct-Pure water system (LabDynamics, Roodepoort, South Africa) provided ultrapure water to use throughout the study. Separations (Randburg, South Africa) supplied Parafilm^®^, Whatman^®^ filter paper, Dulbecco’s Modified Eagle’s Medium (DMEM) with high glucose (HyClone), and Dow Corning^®^ high vacuum grease. Whitehead Scientific (Pty) Ltd. (Cape Town, South Africa) provided Trypan Blue solution (0.4%), L-glutamine (200 mM), penicillin/streptomycin (pen/strep) (10,000 U/mL each), and Trypsin-Versene^®^ (EDTA). Thermo Fisher Scientific (Gibco™, Johannesburg, South Africa) supplied Fetal Bovine Serum (FBS).

### 2.2. Quantification of Ibuprofen

A Shimadzu^®^ Nexera-I LC-2040C 3D Plus system with a gradient pump, column oven (at 22 °C), photodiode array detector, and autosampler injector mechanism was used to validate the HPLC method. A reverse phase Venusil^®^ XBP column (Agela Technologies, Torrance, CA, USA) containing C18(2) silica (150 × 2.1 mm) with a particle size of 5 μm was used. The isocratic system employed a 40% mobile Phase (A) (ultrapure water with 0.1% (*v*/*v*) analytical grade formic acid) and a 60% mobile Phase (B) (chromatography-grade acetonitrile with 0.1% (*v*/*v*) analytical grade formic acid). The total run time was 4.5 min with a flow rate of 0.50 mL/min and a detection wavelength of 224 nm. The injection volume was 10.0 µL. The limit of detection (LOD) and limit of quantification (LOQ) were 0.09 and 0.28 µg/mL, respectively.

### 2.3. Examination of Physicochemical Properties of Ibuprofen

#### 2.3.1. Solubility of Ibuprofen in PBS

Four separate test tubes were filled with 5 mL PBS (pH 7.4). Three of the test tubes were oversaturated with ibuprofen, while the fourth was left to contain only PBS and served as a control. Magnetic stirring rods were added to the test tubes before they were placed in a Grant^®^ JB water bath (Grant Instruments Ltd., Cambridge, UK) equipped with a Variomag^®^ magnetic stirring plate (Variomag, Port Orange, FL, USA) for 24 h at 32 °C, which is the temperature on the surface of human skin [31]. After 24 h, the test tubes were centrifuged at 2600× *g* for 15 min, and 1 mL of supernatant was filtered into HPLC vials using 0.45 µm polytetrafluoroethylene (PTFE) filters and analyzed in duplicate on the HPLC.

#### 2.3.2. Solubility of Ibuprofen in N-Octanol

The same technique discussed to determine solubility in the previous section determined ibuprofen’s solubility in n-octanol; however, n-octanol was utilized instead of PBS. After centrifugation, 1 mL of supernatant was diluted in 10 mL methanol and placed in an ultrasonic water bath to ensure proper dissolution before filtering 1 mL of the solution into HPLC vials.

#### 2.3.3. Octanol-Buffer Distribution Coefficient of Ibuprofen

The shake-flask method determined the octanol-buffer distribution coefficient (log D) value of ibuprofen by co-saturating equal volumes of n-octanol and PBS (pH 7.4) in a separating funnel [32,33]. After 24 h of equilibration, the two phases separated into PBS (pH 7.4) (bottom layer) and n-octanol (top layer), from which 20 mL of the pre-saturated n-octanol was mixed with 49.3 mg ibuprofen, which was calculated according to the solubility value determined previously. The ibuprofen/n-octanol mixture (3 mL) and pre-saturated PBS (pH 7.4) (3 mL) were added to three separate test tubes and rotated for ±12 h in a pre-heated 32 °C shaker water bath, whereafter, they were removed and allowed to separate for 2 h. A micropipette transferred 1 mL from the top and bottom layers, respectively, consisting of n-octanol and PBS (pH 7.4), to 10 mL volumetric flasks, which were diluted to volume with methanol to protect the HPLC. A small volume of each dilution was analyzed on the HPLC, and the log D of ibuprofen was established with a logarithmic ratio of the ibuprofen concentrations detected in the n-octanol and PBS phases.

### 2.4. Formulation of Nano-Drug Delivery Systems

#### 2.4.1. Formulation of a Nano-Emulsion Containing Ibuprofen

After finding an optimal oil concentration and workable surfactant range, three nano-emulsions with different surfactant ratios were prepared, from which the optimized nano-emulsion could be selected. For the oil phase, EPO was pre-heated to ~75 °C on a magnetic hot plate, after which ibuprofen and then Span^®^ 60 were gradually added while stirring with a magnetic stirring bar. Ultrapure water and Tween^®^ 80 were stirred together on a magnetic hot plate and heated to ~75 °C to form the aqueous phase. Once fully dissolved, the oil phase was added to the aqueous phase in drops while continuously stirring for 5 min to create a coarse emulsion [34]. The coarse emulsion was transferred to an ice bath to keep it cool while ultrasonicating (Hielscher Ultrasonic Processor UP200St (Hielscher Ultrasonics, Teltow, Germany)) for 8 min to form the nano-emulsion. The optimized nano-emulsion, containing 5% (*w*/*v*) ibuprofen, was labeled NE, and the formula is given in Table 1.

#### 2.4.2. Formulation of a Nano-Emulgel Containing Ibuprofen

Three nano-emulgels were formulated using the formula of the optimized nano-emulsion but included different concentrations of the gelling agent, Carbopol^®^ Ultrez 20. Preparation of the oil phase was the same as explained for the nano-emulsions, while Carbopol^®^ Ultrez 20 was gradually added to the aqueous phase after the Tween^®^ 80 dissolved. The aqueous phase was stirred for 2 min to neutralize Carbopol^®^ Ultrez 20 and eliminate air bubbles. The overhead stirrer was set to 2000 rpm while the oil phase was added to the aqueous phase in a dropwise manner, and stirring continued for 15 min to produce a coarse emulgel. Lastly, the nano-emulgel was ultrasonicated (Hielscher Ultrasonic Processor UP200St (Hielscher Ultrasonics, Teltow, Germany)) for 8 min while being kept cool in an ice bucket [34]. The optimized nano-emulgel, containing 5% (*w*/*v*) ibuprofen, was labeled NEG (the formula is given in Table 1).

#### 2.4.3. Formulation of a Colloidal Suspension Containing Ibuprofen-Loaded Nanoparticles

Ibuprofen-loaded nanoparticles were formulated in small batches using the emulsion-solvent evaporation method, which required an aqueous and organic phase [35]. The aqueous phase was prepared by stirring PVA in ultrapure water on a magnetic hot plate at ~80 °C until the PVA dissolved. Equal quantities of ibuprofen and PCL were dissolved in DCM and stirred for 20 min, which prepared the organic phase. The aqueous phase was kept on ice while the organic phase was added dropwise with a syringe whilst ultrasonicating. A milky solution resulted, which was transferred to a rotary evaporator (Rotavapor^®^ Büchi RII (Buchi, Basel, Sitzerland)) to evaporate the DCM at 40 °C. The solution was centrifuged at 10,000× *g* for 30 min, whereafter the supernatant was removed, and the remaining pellet was washed once using ultrapure water to eliminate any remnant residue. The remaining pellet was re-dispersed in ultrapure water and placed in an ultrasonic bath for 15 min. Prior to storing the dispersion in a −80 °C freezer for 12 h, a cryoprotectant solution (10 mg/mL sucrose) was added to the dispersion in a 1:2 ratio (cryoprotectant:dispersion). Finally, the tubes were freeze-dried for 72 h; thereafter, they remained in a desiccator to remove excess moisture and keep the nanoparticles dry. The dry nanoparticles were labeled NP. The correct quantity of NPs was weighed and suspended in PBS (pH 7.4) to produce a colloidal suspension, which was labeled CS [36,37]. Due to severe precipitation at a 5.0% (*w*/*v*) ibuprofen concentration, CS had to be formulated at 2.3% (*w*/*v*) ibuprofen concentration. The formulas for NP and CS are given in Table 1.

### 2.5. Characterization of the Drug Delivery Systems

All the drug delivery systems were evaluated in terms of appearance, pH, droplet/particle size, and zeta potential. System-specific characteristics were also evaluated, such as viscosity for NE and NEG, morphology for NE, and X-ray powder diffraction (XRPD) for NP.

#### 2.5.1. Visual Inspection

All the developed drug delivery systems were visually inspected for evident indications of sedimentation, coalescence, creaming, or flocculation.

#### 2.5.2. pH

A Mettler Toledo^®^ pH meter (Mettler Toledo, Columbus, OH, USA) equipped with a Mettler Toledo^®^ InLab^®^ 410 electrode was used for the measurements. The pH meter underwent calibration before each new measurement at pH values of 4, 7, and 10; thereafter, three independent measurements were taken by inserting the electrode into the drug delivery system [38].

#### 2.5.3. Droplet/Particle Size and Distribution

A Malvern Zetasizer Nano ZS (Malvern Instruments, Malvern, Worcestershire, UK) determined the droplet/particle size and distribution of the drug delivery systems. Samples of NE and NEG were prepared by diluting one drop with ultrapure water in a 100 mL volumetric flask. The dilutions were placed in an ultrasonic bath to ensure adequate mixing; thereafter, 2 mL of each dilution was transferred into a polystyrene cuvette using a syringe. For CS, 2 mL of the dispersion was transferred directly into a cuvette after adequate mixing in an ultrasonic bath. Samples were analyzed in triplicate to determine an average droplet/particle size and polydispersity index (PDI) value [37,38].

#### 2.5.4. Zeta-Potential

Sample preparation and zeta-potential measurements used similar methods and equipment as explained for size and distribution measurements, except transparent disposable folded capillary zeta-cells were used to take the measurements in triplicate [37,38].

#### 2.5.5. Viscosity

The viscosity of NE and NEG was measured with a Brookfield viscometer DV2T LV Ultra (Middleboro, MA, USA) coupled to a thermostatic water bath. An hour before the test, the drug delivery systems were placed in a water bath (~25 °C). Each system was measured using a pre-determined spindle. NE used a TB-92 spindle, while NEG used a TE-95 spindle. The Rheocalc T1.2.19 software programmed speeds of 120 rpm for NE and 10 rpm for NEG, and viscosity was measured in centipoise (cP) at multiple points (10 s intervals) for 1 min [38].

#### 2.5.6. Morphology

The morphology of NE was determined with a FEI Tecnai G2 20S-Twin 200 kV high-resolution transmission electron microscope (HRTEM) (Brno, Czech Republic, EU) equipped with an Oxford INCA X-Sight EDS System. Sample preparation diluted a small volume of NE in ultrapure water. The dilution was placed on a microscopic carbon-coated 300 mesh copper grid and left to dry for 10 min; thereafter, the grid was stained with osmium tetroxide, and after allowing another 20–30 min for drying, the grid was analyzed at a voltage of ±200 kV. A Gatan bottom-mount camera with digital micrograph software (DigitalMicrograph 3.5) captured micrographs at different magnifications [38].

#### 2.5.7. XRPD Analysis

XRPD patterns were generated with a PANalytical Empyrean diffractometer (PANalytical, Almelo, The Netherlands) with a PIXcel3D detector. Each powder was spread on a zero-background sample holder equally, and analysis conditions were set as target: Cu; voltage: 45 kV; current: 40 mA; wavelength (λ): 1.5406 Å, and step size: 0.01°.

### 2.6. Diffusion Experiments

Membrane release, in vitro skin diffusion, and tape stripping studies investigated the developed drug delivery systems and a MP with regards to API release through membranes, as well as transdermal and topical delivery of API through dermatomed human skin. Drug delivery systems were freshly prepared before each diffusion study. The donor and receptor chambers of the Franz cells were separated by the relevant membrane, depending on the type of study. The donor and receptor chambers of the Franz cells were greased with vacuum grease, assembled, and secured with a horseshoe clamp. Each receptor chamber contained a magnetic stirring bar to stir the receptor phase continually. PBS (pH 7.4) and the receptor phases of the fully assembled Franz cells were submerged in a water bath equipped with a magnetic stirring plate and monitored to maintain a constant temperature (~37 °C), which mimics in vivo conditions. With a syringe, the receptor chamber of each Franz cell (2 mL) was filled with PBS (pH 7.4), and care was taken to prevent the formation of air bubbles during filling. A second water bath was used to pre-heat the drug delivery systems (~32 °C), which mimics external skin temperature, from which a volume of 1 mL was added to the donor phase. The top of the donor chambers was sealed with Parafilm^®^ and secured with a marked plastic lid to indicate the order of filling. For each membrane release and in vitro skin diffusion study, twelve Franz cells were utilized, where ten Franz cells carried one particular drug delivery system, and the remaining two Franz cells carried the corresponding control placebos. Extraction of the entire receptor phase through the sampling port occurred at specific time intervals for transfer to HPLC vials for analysis [38].

#### 2.6.1. Membrane Release Studies

During membrane release studies, the donor and receptor chambers of the Franz cells were separated by polyvinylidene fluoride (PVDF; 0.45 µm) synthetic membranes. Membrane release studies were conducted over a 6 h period, and the receptor phases of all the Franz cells were extracted and refilled sequentially with PBS (pH 7.4) hourly. The resulting samples were analyzed with HPLC.

#### 2.6.2. Skin Preparation

Ethical authorization (Ethics no: NWU-00111-17-A1-11) from the North-West University Health Research Ethics Committee was obtained before the in vitro skin diffusion studies commenced. To reduce variations, this study used the abdominal skin of Caucasian females obtained with prior informed consent from abdominoplasty donors. A dermatological examination ensured that no abnormalities were present that could affect the study’s outcomes. Dermatomed skin samples with a thickness of ~400 μm were procured with a Dermatome™ (Zimmer 201 TDS, Warsaw, IN, USA). The samples were placed on Whatman^®^ filter paper, wrapped in aluminum foil, sealed in airtight plastic bags, and kept in the freezer at −20 °C. When required for studies, the skin was thawed and cut into circular pieces to fit between the donor and receptor chambers of the Franz cells.

#### 2.6.3. In Vitro Skin Diffusion Studies

During the in vitro skin diffusion studies, the donor and receptor chambers of the Franz cells were separated by the prepared dermatomed skin samples with the stratum corneum facing upwards to the donor phase. In vitro skin diffusion studies were conducted over a 12 h period. The receptor phases of all the Franz cells were extracted and refilled sequentially every 20 min for the first 2 h, then every 2 h for the remaining 10 h. The resulting samples were analyzed with HPLC.

#### 2.6.4. Tape Stripping

The amount of API retained in the stratum corneum-epidermis (SCE) and epidermis-dermis (ED) after in vitro skin diffusion studies was determined with tape stripping to provide an indication of the amount of API that was topically delivered. A paper towel was used to gently dab the skin samples and remove the remaining excess formulation. The skin sample from each of the 12 Franz cells was tape stripped using 16 pieces of 3 M Scotch^®^ Magic™ tape, but the first strip of tape was discarded to avoid contamination. After pressing the remaining 15 tape strips against the diffusion area, they were placed into polytops containing 5 mL methanol, whereafter, the skin had a glistening appearance indicating the removal of the SCE. The residual skin sample, with only the ED remaining, was cut into pieces and placed in polytops containing 5 mL methanol. The polytops were stored in a refrigerator overnight (±12 h) at ~4 °C, whereafter, the samples were filtered into HPLC vials and analyzed.

### 2.7. In Vitro Cytotoxicity Assays

#### 2.7.1. Cell Culturing Conditions

The HaCaT cells were cultured in flasks containing growth medium, which consisted of high-glucose DMEM with 1% NEAA, 1% pen/strep, 10% FBS, and L-glutamine (2.0 mM) as supplements. An ESCO Cell Culture CO_2_ incubator (Esco Technologies, Inc., St. Louis, MO, USA) incubated the flasks at ~37 °C, 5% CO_2_, and 95% humidity. The flasks were checked under an inverted light microscope every 48 h for undesirable bacterial growth and confluency estimation before replacing the depleted cell culture medium. At 80–90% confluency, the cells were sub-cultured using trypsinization (EDTA), whereafter, the Trypan blue staining method was employed to measure the concentration of viable cells in the cell suspension. A cell suspension with the desired concentration of 75,000 cells/mL for cytotoxicity studies was prepared, whereafter, 200 µL of the cell suspension was added to the wells of a 96-well plate, which obtained a density of 15,000 cells per well. The 96-well plates were incubated for 24 h at ~37 °C, 95% humidity, and 5% CO_2_ to allow cell recovery.

After incubating the HaCaT cells for recovery, they were treated with a concentration range of the following stock solutions for 12 h: ibuprofen (200–500 µg/mL), NE (200–500 µg/mL), PNE (200–500 µg/mL), CS (50–350 µg/mL). Ibuprofen was diluted in methanol and then mixed with the culture medium to obtain a stock solution, while the stock solutions of NE, PNE, and CS only required the addition of the culture medium to obtain the desired treatment concentrations. The stock solution of ibuprofen did not contain methanol concentrations above 5%.

#### 2.7.2. MTT-Assay

The MTT assay was conducted using a method adapted from Fouché et al. [39]. After the 12 h incubation period, the two 96-well plates were removed from the incubator and aspirated. The wells were washed in duplicate with 100 µL phosphate-buffered saline; thereafter, 200 µL of MTT solution (0.5 mg/mL in non-supplemented DMEM) was added to each well. The 96-well plates with light-sensitive MTT were covered with aluminum foil and incubated for 4 h at ~37 °C, 5% CO_2_, and 95% humidity. After exposure, the MTT solution was aspirated, 200 µL DMSO was added to each well, and the aluminum-wrapped plate was placed on a microplate shaker for 1 h to solubilize the MTT-formazan crystals. The plates were placed in a SpectraMax^®^ Paradigm^®^ multi-mode microplate reader (Molecular devices, San Jose, CA, USA) to measure the absorbance with SoftMax^®^ Pro 6.2.1 software at 560 nm cell and 630 nm background signals. Three rows on the 96-well plates served as the control groups, which included an untreated row, a DMSO blank row, and a dead cell row (cells treated for 10–15 min with 200 µL Triton™ X-100 (0.2% in phosphate-buffered saline)).

#### 2.7.3. NR-Assay

An NR assay that included some modifications was employed [40]. After incubation, the wells of the 96-well plates were aspirated and washed in duplicate with 100 µL phosphate-buffered saline. Each well received 200 µL NR solution (0.33% NR stock in non-supplemented DMEM, filtered with a 0.45 µm syringe filter). The plates were covered with aluminum foil and incubated for 2 h at ~37 °C, 5% CO_2_, and 95% humidity. The wells were aspirated with NR solution and rinsed with 100 µL fixative (1% calcium chloride in 0.5% formaldehyde), which anchored the cells. A volume of 150 µL solubilization solution (1.0% acetic acid in 50% ethanol) was added to the wells; thereafter, the 96-well plate was wrapped in aluminum foil and placed on a microplate shaker for 10 min. The absorbance was measured at 540 nm cell and 690 nm background signals using the same equipment mentioned for the MTT assay. Control groups were identical to those of the MTT assay, except a solubilization blank row replaced the DMSO blank row.

#### 2.7.4. Calculation of the IC50 Values

The half-maximal inhibitory concentration (IC50 value) of each treatment group was determined from the obtained in vitro cytotoxicity data. The following equation calculated the %viable cells [39,41]:

%Viable cells = ((ΔSample − Δblank)/(ΔUntreated control − Δblank)) × 100(1)

The absorbance for the MTT-assay (560–630 nm) and NR-assay (540–690 nm) was measured at different wavelengths, and Δ represents the difference between these values. The %viable cells at each concentration were subtracted from the initial %viable cells (100%) to calculate the %inhibited cells. Regression analysis calculated the IC50 value (x-value), where the y-value was set to 50% (inhibition), m was the slope, and c was the y-intercept [42,43].

### 2.8. Statistical Data Analysis

Descriptive and interferential statistics used STATISTICA^®^ 13.3 (StatSoft, TIBCO^®^ Software Inc., Palo Alto, CA, USA) to analyze the data obtained from membrane release and in vitro skin diffusion studies. Boxplots are descriptive statistics that visually convey the distribution of the data by summarizing the data of the lower quartile (Q1 or 25th percentile), median (Q2 or 50th percentile), upper quartile (Q3 or 75th percentile), and extreme values [44]. Inferential statistics included one-way analysis of variance (ANOVA) to analyze the data from the membrane release and in vitro skin diffusion studies, while two-way ANOVA analyzed the data from the tape stripping studies; however, the assumptions of ANOVA were violated, and as a result, log transformations were implemented as remedial measures [45]. The ANOVA omnibus F-test determined a significant main effect or interaction effect, while a Bonferroni post-hoc test drew comparisons between multiple data points, such as the average concentration and flux values of the various drug delivery systems. Statistical significance is expressed by a *p*-value between 0–1, where a *p*-value ≤ 0.05 rejects the null hypothesis and is regarded as statistically significant [46].

## 3. Results and Discussion

### 3.1. Examination of Physicochemical Properties of Ibuprofen

#### 3.1.1. Solubility of Ibuprofen in PBS and N-Octanol

An API is regarded as suitable for transdermal delivery if its aqueous solubility is greater than 1 mg/mL [47]. Laboratory testing calculated the aqueous solubility of ibuprofen as 0.651 mg/mL; therefore, API diffusion through the skin might be hindered since low aqueous solubility is not ideal for transdermal delivery. Furthermore, ibuprofen’s solubility in n-octanol was determined as 2.462 ± 0.041 mg/mL.

#### 3.1.2. Log D of Ibuprofen

According to the literature, APIs with an octanol-water partition coefficient (log P) < −1 possess hydrophilic properties and would, therefore, be unfavorable for transdermal delivery since the API would have difficulty permeating through the lipophilic stratum corneum, while APIs that possess a log P > −1 have lipophilic properties and are better candidates for transdermal delivery [48,49]. Ideally, an API should have both lipophilic and hydrophilic characteristics with a log D of 1–3 [50]. Calculations using the concentrations of ibuprofen detected in PBS and n-octanol determined the log D of ibuprofen as 1.343 ± 0.019 at a pH of 7.4. Since ibuprofen obtained a higher log D value, it was determined that the API shows potential for transdermal drug delivery [51].

### 3.2. Characterization of the Drug Delivery Systems

**Figure 1:**
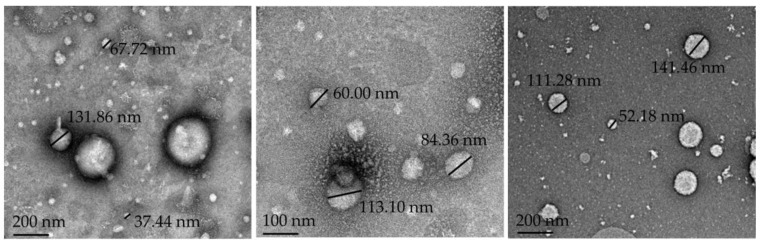
TEM micrographs of oil droplets in NE with scale bars that indicate magnification.

**Figure 2:**
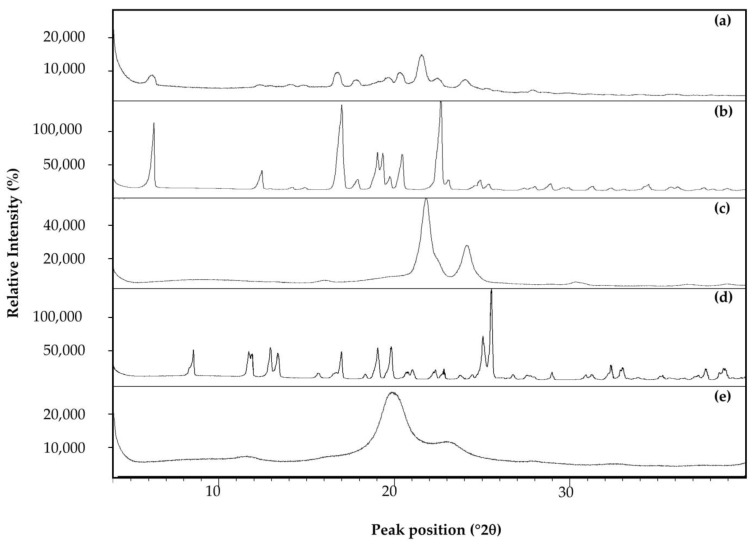
An XRPD overlay for: (**a**) the ibuprofen-loaded nanoparticles, (**b**) ibuprofen, (**c**) PCL, (**d**) sucrose and (**e**) PVA.

Table 2 presents a summary of the results obtained from characterization tests of the developed drug delivery systems. Figure 1 shows the TEM micrographs of NE, while Figure 2 displays the XRPD results.

After formulation, the drug delivery systems were visually examined for physical instabilities, such as sedimentation, coalescence, creaming, or flocculation. NE appeared homogenous with a milky white color and no visible signs of instabilities. A clear difference was seen in the viscosity of NEG, which presented as a thick, homogenous white gel with no visible signs of instability. The CS presented with a low viscosity and appeared watery with no initial signs of instability; however, after a few hours, some precipitation occurred.

The micrographs of the NE (Figure 1) reveal the morphology of the oil droplets dispersed in the water phase, which confirms round droplets with sizes < 200 nm that comply with the requirements of a nano-emulsion drug delivery system.

The pH values of the drug delivery systems were measured since transdermal formulations are required to have pH values between 3 and 9; otherwise, it could negatively affect the integrity and permeability of the skin [52]. All the formulations obtained acceptable pH values and should, therefore, be safe for topical application.

Transdermal drug delivery is more effective with smaller particle sizes, and to be classified as a nano-drug delivery system, particle sizes should be <500 nm. All the drug delivery systems, NE, NEG, and CS, obtained particle sizes < 200 nm and should, therefore, effectively deliver APIs transdermally [53,54].

Lower PDI values suggest a more stable monodispersed or homogenous system, while higher PDI values suggest a less stable polydisperse system [55,56]. The PDI results of all the drug delivery systems show lower PDI values closer to zero, confirming they were monodispersed and more stable [56].

A zeta potential of more than 30 mV or less than −30 mV indicates that the drug delivery system will stay stable over longer periods [57,58,59]. NE and NEG displayed negative zeta-potential values below −30 mV, while CS fell just short of the requirement, and this result corresponds with the precipitation seen in CS after a few hours, with a possible cause being that the density of the particles in CS was greater than that of the dispersant [60].

Data obtained from viscosity tests showed that CS had no viscosity, NE had low viscosity, and NEG had high viscosity. NEG was developed with the purpose of increased viscosity since higher viscosity may enhance skin penetration and promote skin adherence to make transdermal application more effortless [57,61].

The diffraction patterns obtained from XRPD analysis showed an absence of high-intensity diffraction peaks for the ibuprofen-loaded nanoparticles, PCL and PVA, indicating an amorphous nature, while the diffractograms of ibuprofen and sucrose stand in stark contrast, displaying multiple high-intensity diffraction peaks, which indicate a crystalline nature (Figure 2).

### 3.3. Diffusion Experiments

The data obtained from diffusion studies and tape stripping will be discussed using median values since they are more reliable and not influenced by outliers [62]. In addition, boxplots visually conveyed the distribution of the data.

#### 3.3.1. Membrane Release Studies

**Figure 3:**
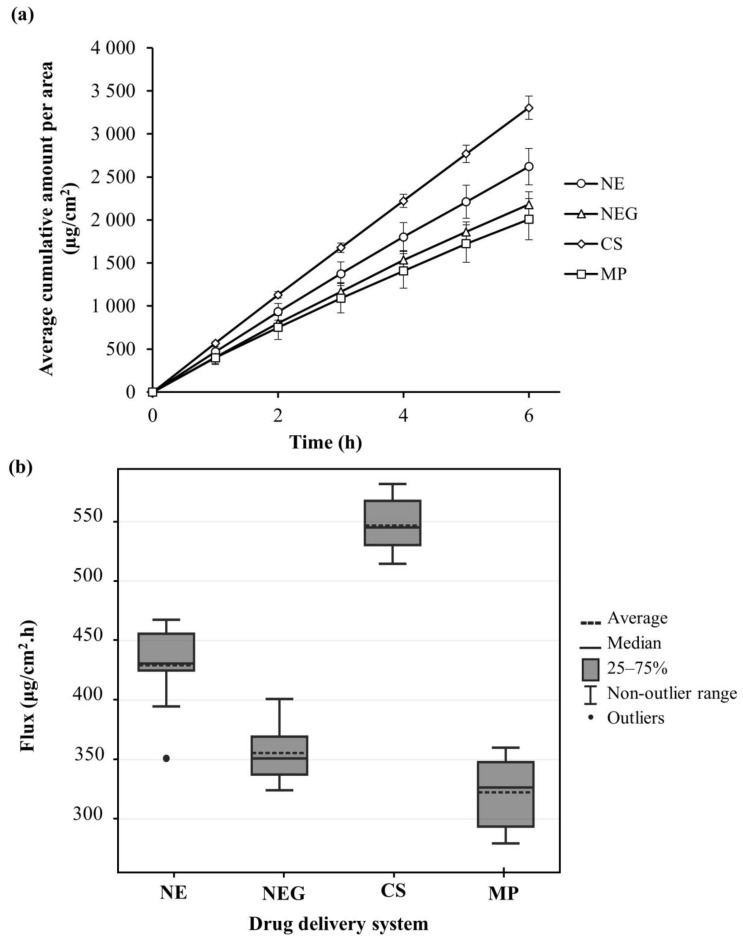
(**a**) Average cumulative amount of ibuprofen that diffused per area (μg/cm^2^) of membrane from each drug delivery system over a period of 12 h and (**b**) boxplot indicating the average and median flux (μg/cm^2^.h) for each drug delivery system during membrane release studies.

Table 3 and Figure 3 summarize the results from the membrane release studies. All the developed systems (NE, NEG, and CS) obtained higher drug release than the market-related product (MP). The CS and NE had the lowest viscosity; hence, these findings agree with previous research, indicating that systems with lower viscosity result in improved API release [56]. The increased surface-to-volume ratio associated with smaller molecules could also result in enhanced drug release [26,63]. However, CS had the highest drug release, even though it only contained 2.3% (*w*/*v*) ibuprofen compared to the rest of the drug delivery systems that contained 5% (*w*/*v*) ibuprofen, which made it an exciting prospect for the in vitro skin diffusion studies. Over the entire 6 h period, it was observable that all the drug delivery systems effectively released ibuprofen, allowing in vitro skin diffusion studies to proceed.

One-way ANOVA established that a statistically significant difference exists between the average flux values of the drug delivery systems. The Bonferroni posthoc test further determined the specific pairs of drug delivery systems that showed a statistically significant difference, and all the pairwise comparisons between the drug delivery vehicles showed highly significant statistical differences (*p* < 0.001) except for NE and MP (*p* = 0.091), which showed no statistically significant difference.

#### 3.3.2. In Vitro Skin Diffusion Studies

**Figure 4:**
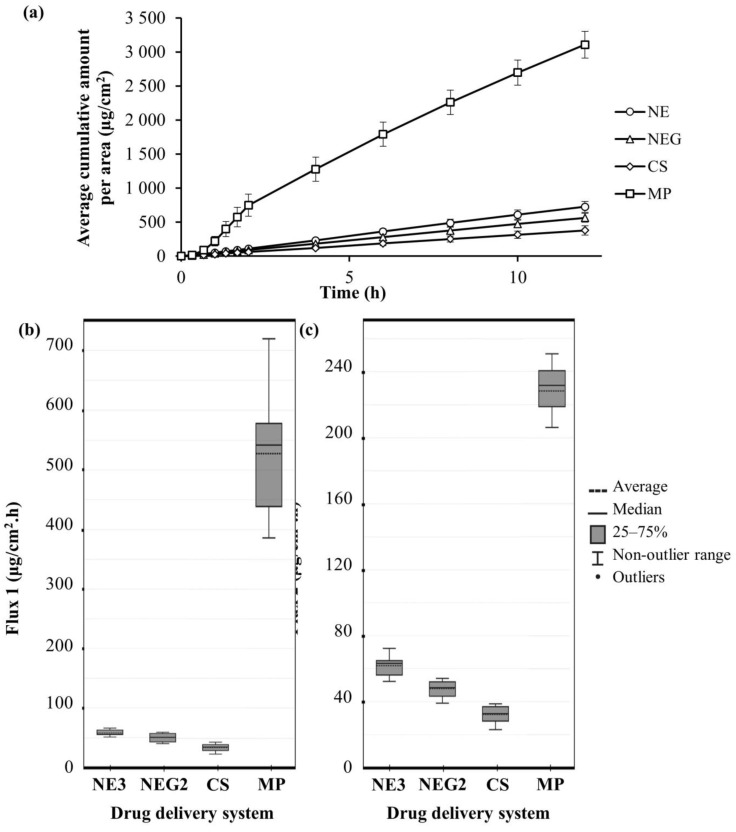
(**a**) Average cumulative amount of ibuprofen that diffused per area (μg/cm^2^) of skin from each drug delivery system over a period of 12 h and (**b**) boxplot indicating the average and median flux 1 (μg/cm^2^.h) taken from 1–2 h and (**c**) flux 2 (μg/cm^2^.h) taken from 4–12 h for each drug delivery system during in vitro skin diffusion studies.

Table 4 and Figure 4 summarize the results from in vitro skin diffusion studies, which obtained two median flux values, where flux 1 was taken from 1–2 h and flux 2 from 4–12 h. Flux 1 and flux 2 followed an identical pattern, with MP obtaining the highest median flux value, followed by NE, NEG, and then CS.

It was evident that the MP achieved diffusion that was exponentially higher in comparison to NE, NEG, and CS. The MP’s in vitro results stand in stark contrast with the low release it showed during membrane release studies. This, however, is likely due to the inclusion of 3% levomenthol and a high amount of ethanol as excipients, which act as potent penetration enhancers that disrupt the lipid packing of the stratum corneum and increase the drug uptake of the stratum corneum, respectively [64,65]. Due to the absence of a stratum corneum in membranes, the membrane release studies did not reveal the excellent penetration-enhancing effects of levomenthol and ethanol.

For the NE and CS, constant API diffusion was observed over the entire 12 h period, while the MP exhibited slow API diffusion for 30 min, followed by exponential API diffusion up to 2 h, and thereafter, the API diffusion became constant. NEG also exhibited slow initial API diffusion that became constant after 30 min. Goebel et al. [66] reported that semi-solid drug delivery systems, such as MP and NEG, exhibit two distinct fluxes and require 2–3 h to attain steady-state flux.

When comparing flux 1 and flux 2 for only NE, NEG, and CS, the observation is that NE obtained the highest median flux values followed by NEG then, CS, indicating that NE had the quickest diffusion. However, as discussed, the CS was formulated at a lower concentration of 2.3% ibuprofen compared to NE and NEG with a 5% ibuprofen concentration; therefore, lower flux values for CS were expected. In this case, the average %diffused gives a more accurate representation since it accounts for CS’s lower API concentration, and according to this value, the CS obtained the highest diffusion followed by NE, then NEG. It is also noteworthy to mention that the CS contained no penetration enhancers, gelling agents, or stabilizers and acquired the best skin diffusion. The NE obtained better results than NEG during the in vitro skin diffusion studies, which can be explained by scientific literature [67], which states that nano-emulsions (NE) exhibit greater skin diffusion and steady-state fluxes than semi-solid formulations (NEG).

Literature [22,68] also emphasizes that smaller droplet sizes, such as those of the nano-drug delivery systems (NE, NEG, and CS), are capable of more rapid API diffusion over the various epidermal layers. Therefore, it would be beneficial to include a similar amount of levomenthol during the development of future drug delivery systems to directly compare the developed drug delivery systems and the market-related product (MP).

The therapeutic blood concentration for ibuprofen ranges between 10 and 50 µg/mL [69]. The average concentration of ibuprofen that diffused during in vitro skin diffusion studies (Table 4) is much higher than the amount necessary to reach therapeutic blood concentrations. However, a drug’s in vitro potency does not predict the in vivo efficacy due in part to the drug’s pharmacokinetic features, according to Gleeson et al. [70]. Safety of use for the market-related product (MP) has already been established, and it obtained exponentially higher concentrations compared to the developed drug delivery systems (NE, NEG, and CS); therefore, it can be concluded that all the drug delivery systems did reach blood concentrations high enough to have therapeutic effects during the in vitro skin diffusion studies and should also reach therapeutic blood concentrations to some extent during in vivo studies. The conclusion was that all the systems (NE, NEG, CS, and MP) delivered ibuprofen through the skin continuously over the 12 h period.

The p-values obtained from the one-way ANOVA F-test were <0.001 for flux 1 and 2, which indicated highly significant statistical differences. The Bonferroni posthoc test further determined which specific pairs of drug delivery systems showed a statistically significant difference. For flux 1, all the pairwise comparisons showed highly significant statistical differences (*p* < 0.001), except for NE and NEG (*p* = 0.709), which showed no statistically significant difference. For flux 2, all the pairwise comparisons showed highly significant statistical differences (*p* < 0.001), indicating that a relationship exists between the pairs and that the data did not occur due to chance [71].

#### 3.3.3. Tape Stripping

**Figure 5:**
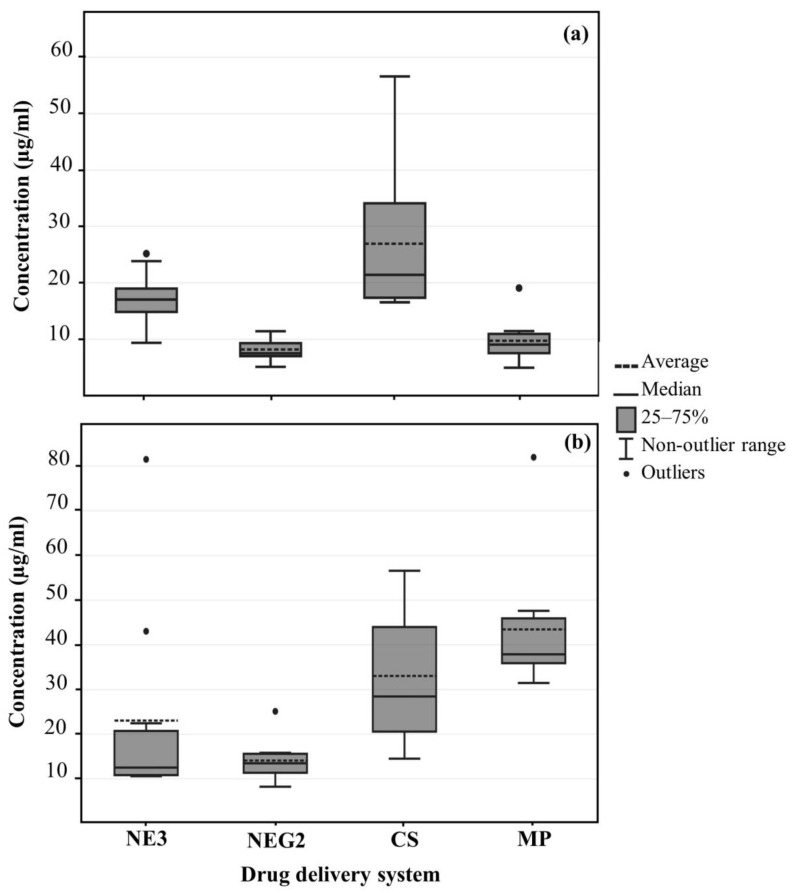
Boxplot of the average and median ibuprofen concentrations (µg/mL) in (**a**) the SCE and (**b**) the ED of the different drug delivery systems after a 12 h in vitro skin diffusion study.

Table 5 and the boxplots in Figure 5 summarize the data collected by means of tape stripping. Among the various drug delivery systems, the highest residual concentration of ibuprofen within the SCE (topical delivery) was detected from CS, followed by NE, MP, and lastly, NEG, while MP obtained the highest concentration of ibuprofen in the ED, followed by CS, NEG and lastly, NE.

Literature suggests that polymeric nanoparticles <200 nm penetrate the skin through hair follicles using the transappendageal pathway, which accounts for less than 0.1% of the skin’s surface [72,73]. Once the hair follicles become oversaturated, CS would accumulate in skin furrows and hair follicles, sequentially increasing the local concentration of API that can readily diffuse to viable skin layers. Therefore, the large amount of API that resided in the SCE and ED from CS is due to its accumulation in the skin furrows and hair follicles.

Nano-emulsions and nano-emulgels containing additional penetration enhancers are expected to traverse the lipid matrix of the SCE more easily, as they are lipophilic in nature and primarily follow the intercellular pathways [74,75]. Therefore, less API from NE and NEG resided in the SCE and ED, as the API could successfully permeate through the SCE and ED into deeper skin layers.

It is notable that compared to the SCE, MP obtained a much higher concentration of API in the ED. The ethanol in MP would have increased the drug uptake ability of the stratum corneum, while the levomenthol disrupted the lipid packing of the stratum corneum, making permeation into the ED much easier [63,64]. Therefore, it seems that the API permeated into the ED faster than it could diffuse into the receptor phase during the in vitro skin diffusion studies, which created a drug load in the ED.

Tape stripping data offered two variables, namely the drug delivery systems and the tape stripping data; therefore, a two-way ANOVA was employed to determine statistically significant differences between the two variables and the effect on the average concentrations. The F-test obtained highly significant statistical differences (*p* < 0.001) for the drug delivery vehicles and tape stripping data. The F-test p-value for the interaction effect between the two variables also indicated highly statistically significant differences (*p* < 0.001). The interaction effect is further elaborated by pairwise comparisons of the average concentrations of the various drug delivery systems within the SCE and ED groups by using a Bonferroni post-hoc test, which indicated no statistically significant differences between the following groups within the SCE: NE and NEG, NE and MP, NEG and MP, or within the ED: NE and NEG, CS and MP. Comparison made between the concentrations in SCE and ED within each delivery system revealed that NE and NEG showed no statistically significant difference in the average concentration of ibuprofen between the SCE and ED. All other pairwise comparisons obtained significant statistical differences (*p* < 0.05) [71].

### 3.4. In Vitro Cytotoxicity Findings

**Figure 6:**
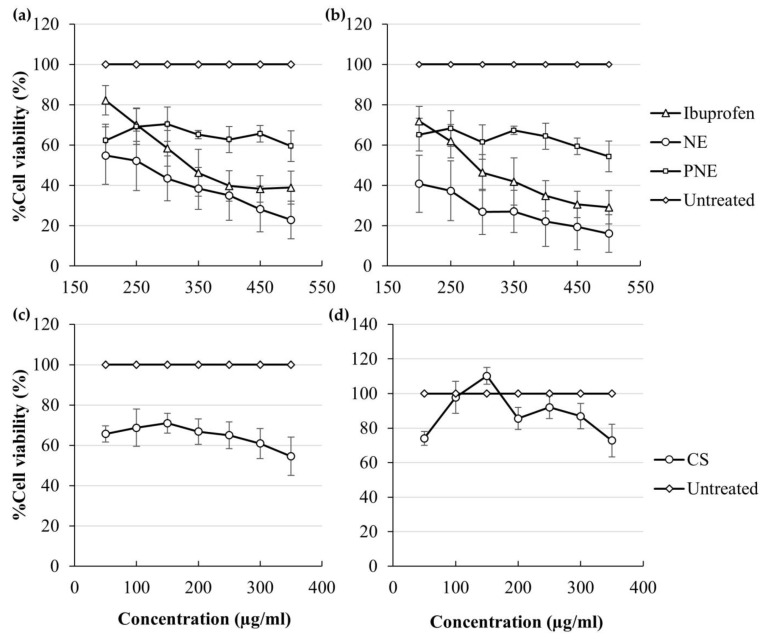
%Cell viability of HaCaT cells after 12 h exposure to different concentration ranges of ibuprofen, NE, and PNE during the (**a**) MTT assay and (**b**) NR assay and for CS during the (**c**) MTT assay and (**d**) NR assay.

Table 6 lists the IC50 values of ibuprofen NE, PNE, and CS acquired from the MTT and NR assays on HaCaT cells. Figure 6 displays the regression curves obtained from the in vitro cytotoxicity studies of ibuprofen, NE, PNE, and CS.

NE obtained the lowest IC50 values for both assays, indicating the highest cytotoxic ability and potency since a lower concentration is required to reduce %cell viability to 50%. The NE was followed by ibuprofen, then CS, and lastly, PNE, which had the lowest IC50 value and the least cytotoxic ability.

The %cell viability also indicates the grade of cytotoxicity of a treatment group according to categories, where a %cell viability <40% indicates strong cytotoxic effects, 40–60% indicates moderate toxicity, 60–80% indicates weak cytotoxicity, and >80% indicates no cytotoxicity [76]. In the MTT assay, ibuprofen and NE showed strong cytotoxic effects at their highest concentrations, while PNE and CS showed moderate cytotoxicity at their highest concentrations. The NR assay also revealed that ibuprofen and NE showed strong cytotoxic effects, PNE showed moderate cytotoxic effects, and CS showed weak cytotoxic effects. However, consideration should be given to the fact that the assays measured %cell viability over a wide concentration range at concentrations much higher than the actual concentrations that diffused from the drug delivery systems during in vitro skin diffusion studies. When comparing the actual concentrations that diffused, the conclusion is that minimal cytotoxic effects should occur.

## 4. Conclusions

All of the drug delivery systems were successfully developed to contain ibuprofen and effectively permeated through the skin to distribute ibuprofen systemically at concentrations that have been shown to be minimally cytotoxic. For future reference, levomenthol can be incorporated into the drug delivery systems to enhance API permeation and for a more accurate comparison to the market-related product.

## Figures and Tables

**Table 1:** Formulas utilized in the preparation of different drug delivery systems.

Phase	Excipients	Drug Delivery Vehicle (% *w*/*v*)			
		NE	NEG	NP	CS
Oil	Ibuprofen	5.00	5.00	–	**
	Evening primrose oil	20.00	20.00	–	–
	Span^®^ 60	4.00	4.00	–	–
Aqueous	Ultrapure water	68.00	67.40	74.24	–
	Tween^®^ 80	3.00	3.00	–	–
	Carbopol^®^ Ultrez 20	–	0.60	–	–
	PVA	–	–	0.74	–
	PBS (pH 7.4)	–	–	–	87.83
Cryoprotectant	Sucrose	–	–	0.03	–
Organic *	Ibuprofen	–	–	0.12	–
	PCL	–	–	0.12	–
	DCM	–	–	24.75	–
Solid *	Ibuprofen-loaded NPs	–	–	–	12.17

NE—nano-emulsion; NEG—nano-emulgel; NP—nanoparticles; CS—colloidal suspension. * Instead of an oil phase, the NP has an organic phase, while the CS has a solid phase; ibuprofen was included in these phases. ** CS was formulated at an ibuprofen concentration of 2.3% (*w*/*v*).

**Table 2:** Results from the characterization tests conducted on each drug delivery system.

	NE	NEG	CS
Particle/droplet size (nm)	92.807 ± 0.732	48.230 ± 0.803	166.433 ± 0.929
PDI	0.177 ± 0.003	0.311 ± 0.250	0.279 ± 0.014
Zeta-potential (mV)	−33.367 ± 0.987	−36.400 ± 0.500	−25.433 ± 0.651
pH	5.092 ± 0.016	5.054 ± 0.004	6.607 ± 0.003
Viscosity (cP)	54 ± 10	38 738 ± 1 069	-

NE—nano-emulsion; NEG—nano-emulgel; CS—colloidal suspension.

**Table 3:** Average %released and average and median flux (μg/cm^2^.h) values of the respective drug delivery systems over a 6 h period during membrane release studies (*n* = number of Franz cells used).

	NE	NEG	CS	MP
*n*	10	10	10	10
Average %released (%)	2.812 ± 0.227	2.339 ± 0.159	7.862 ± 0.325	2.159 ± 0.257
Average flux (μg/cm^2^.h)	429.26 ± 34.919	355.46 ± 24.68	546.76 ± 23.61	322.38 ± 31.314
Median flux (μg/cm^2^.h)	430.715	350.925	545.165	326.460

NE—nano-emulsion; NEG—nano-emulgel; CS—colloidal suspension; MP—market-related product.

**Table 4:** Average %diffused, as well as the average and median flux (μg/cm^2^.h) values of both fluxes 1 and 2 over a period of 12 h, with flux 1 taken from 1–2 h and flux 2 taken from 4–12 h during in vitro skin diffusion studies (*n* = number of Franz cells used).

	NE	NEG	CS	MP
*n*	10	10	10	9
Average concentration diffused (µg/mL)	389.31 ± 41.95	302.64 ± 35.30	202.93 ± 35.70	1670.37 ± 104.61
Average %diffused (%)	0.776 ± 0.08	0.605 ± 0.07	0.870 ± 0.15	3.341 ± 0.21
Average flux 1 (μg/cm^2^.h)	58.28 ± 5.40	47.88 ± 7.48	33.65 ± 6.55	527.48 ± 100.17
Average flux 2 (μg/cm^2^.h)	61.73 ± 6.77	50.22 ± 5.41	32.17 ± 5.49	228.29 ± 14.55
Median flux 1 (μg/cm^2^.h)	56.13	50.75	34.62	541.70
Median flux 2 (μg/cm^2^.h)	63.19	48.41	32.67	231.63

NE—nano-emulsion; NEG—nano-emulgel; CS—colloidal suspension; MP—market-related product.

**Table 5:** Average and median concentrations (μg/mL) of ibuprofen in the SCE and ED after the 12 h in vitro skin diffusion studies.

	NE	NEG	CS	MP
Average concentration in SCE (μg/mL)	17.028 ± 5.12	8.180 ± 2.07	23.233 ± 7.84	9.727 ± 4.02
Average concentration in ED (μg/mL)	23.041 ± 22.60	14.083 ± 4.63	33.036 ± 15.88	43.461 ± 15.51
Median concentration in SCE (μg/mL)	17.04	7.50	19.75	9.05
Median concentration in ED (μg/mL)	12.50	13.47	28.44	37.88

NE—nano-emulsion; NEG—nano-emulgel; CS—colloidal suspension; MP—market-related product; SCE—stratum-corneum epidermis; ED—epidermis-dermis.

**Table 6:** Established IC_50_ values for the various treatment groups by using MTT and NR assays.

Treatment Group	IC_50_ Value (µg/mL)	
	MTT-Assay	NR-Assay
Ibuprofen	372	317
NE	251	70
PNE	1268	730
CS	574	604

NE—nano-emulsion; PNE—placebo of nano-emulsion; CS—colloidal suspension; MTT—methyl thiazolyl tetrazolium; NR—neutral red.

## Data Availability

The datasets generated and/or analyzed during the current research project are available from the corresponding author upon reasonable request.
